# Maternal Smoking During Pregnancy and Risk of Autism Spectrum Disorder in Offspring: A Systematic Review and Meta-Analysis

**DOI:** 10.3390/jcm14238584

**Published:** 2025-12-03

**Authors:** Afroditi Peltekidi, Vaidas Jotautis, Maria Tzitiridou-Chatzopoulou, Vasiliki E. Georgakopoulou, Aikaterini Sousamli, Athina Diamanti, Victoria Vivilaki, Eirini Orovou, Antigoni Sarantaki

**Affiliations:** 1 Department of Midwifery, School of Health & Care Sciences, University of West Attica, Egaleo,12243 Athens, Greece; afroditipeltekidi@gmail.com (A.P.); asousamli@uniwa.gr (A.S.); adiamanti@uniwa.gr (A.D.); vvivilaki@uniwa.gr (V.V.); 2 Faculty of Medicine, Kauno Kolegija Higher Education Institution, Pramonės Av. 20, LT-50468 Kaunas, Lithuania; vaidas.jotautis@kaunokolegija.lt; 3 Department of Midwifery, University of Western Macedonia, 50200 Ptolemaida, Greece; eorovou@uowm.gr; 4 Department of Pathophysiology, Laiko General Hospital, National and Kapodistrian University of Athens, 11527 Athens, Greece; vaso_georgakopoulou@hotmail.com

**Keywords:** autism spectrum disorder, maternal smoking, pregnancy, prenatal exposure

## Abstract

**Background/Objectives**: Autism spectrum disorder (ASD) is a heterogeneous neurodevelopmental condition characterized by persistent social-communication deficits and repetitive behaviors. While genetic factors play a major role, prenatal environmental exposures may also contribute. Maternal smoking during pregnancy is a known risk factor for adverse perinatal outcomes, but its association with ASD remains unclear. **Methods**: We conducted a systematic review and meta-analysis following PRISMA 2020 guidelines. A comprehensive literature search was performed in PubMed, Embase, Web of Science, Scopus, PsycINFO, and Google Scholar up to September 2025. Eligible observational studies evaluated maternal active smoking during pregnancy and ASD diagnosis in offspring. Effect estimates were pooled using a random-effects model and expressed as relative risks (RR) with 95% confidence intervals (CI). Heterogeneity was quantified using I^2^, with subgroup and sensitivity analyses performed. **Results**: Twenty-one studies including several million mother–child pairs met the inclusion criteria. The pooled RR for ASD associated with maternal smoking was 1.01 (95% CI: 0.95–1.08), indicating no significant association. Subgroup and sensitivity analyses confirmed the robustness of the findings, with no evidence of publication bias. **Conclusions**: Maternal smoking during pregnancy does not appear to increase ASD risk in offspring. Nevertheless, smoking cessation remains critical due to established adverse fetal effects.

## 1. Introduction

Autism spectrum disorder (ASD) comprises a group of neurodevelopmental conditions characterized by persistent deficits in social communication and interaction, accompanied by restricted and repetitive patterns of behavior, interests, and activities, with considerable heterogeneity in clinical presentation and co-occurring conditions [1]. Surveillance data indicate that ASD affects approximately 1–2% of children worldwide. In the United States, the Autism and Developmental Disabilities Monitoring (ADDM) Network estimates ASD prevalence and monitors identification timing among children aged 4 and 8 years. In 2022, 16 sites across Arizona, Arkansas, California, Georgia, Indiana, Maryland, Minnesota, Missouri, New Jersey, Pennsylvania, Puerto Rico, Tennessee, Texas, Utah, and Wisconsin conducted ASD surveillance for children who lived in these areas. There was an observed increase in the prevalence of ASD among 8-year-old children, with notably higher rates among Asian/Pacific Islander (A/PI), Black, and Hispanic children compared to their White counterparts. Additionally, children from A/PI, Black, and Hispanic backgrounds with ASD were more likely to have a co-occurring intellectual disability than their White or multiracial peers. Importantly, the early identification of ASD by 48 months of age showed improvement for children born in 2018 relative to those born in 2014 [2]. Although ASD is highly heritable, increasing attention has been directed toward potentially modifiable prenatal factors to elucidate environmental contributions to its etiology.

Maternal smoking during pregnancy is a well-established risk factor for adverse perinatal outcomes, including fetal growth restriction, low birth weight, and preterm birth, with clear dose–response relationships reported across cohorts [3,4,5,6]. Biologically, tobacco smoke constituents, such as nicotine, carbon monoxide, and polycyclic aromatic hydrocarbons, can cross the placenta, disrupt trophoblast function, and alter placental epigenetic regulation [7]. Nicotine, in particular, crosses the placental barrier and can reach fetal concentrations equal to or exceeding maternal levels. Experimental evidence suggests that nicotinic acetylcholine receptor–mediated mechanisms interfere with neurogenesis, neurotransmitter signaling, and synaptogenesis during critical periods of brain development [8].

Epidemiological findings regarding maternal smoking and ASD risk, however, remain inconsistent. A 2015 meta-analysis of 15 observational studies reported a pooled odds ratio (OR) of 1.02 [95% confidence interval (CI) 0.93–1.10], suggesting no overall association between maternal smoking during pregnancy and offspring ASD [9]. In contrast, a 2017 meta-analysis examining population-level smoking metrics identified increased odds in European (and one Asian) populations, implying possible effect modification by geographic or exposure context [10]. More recent investigations applying causal inference frameworks and negative-control analyses have highlighted the potential influence of residual familial and genetic confounding, with most findings not supporting a strong causal relationship [11,12].

Nonetheless, emerging large-scale cohort studies have refined exposure classification and dose–response modeling. For example, a multi-site U.S. cohort reported that heavy maternal smoking (≥20 cigarettes/day) was associated with increased ASD risk, whereas lighter smoking showed weaker and statistically imprecise associations in sibling-comparison analyses [13]. Similarly, biomarker-based studies using maternal cotinine measurements have enhanced exposure accuracy; although these studies have not demonstrated robust associations with ASD diagnoses, potential links with autism-related behavioral traits remain under discussion [14]. Variation across studies may stem from differences in exposure assessment (self-report vs. biomarkers; active vs. environmental tobacco smoke), timing and intensity of exposure, concurrent environmental risk factors (e.g., air pollution), and incomplete control for socioeconomic or psychiatric confounding.

Since the most recent meta-analysis by Jung et al. in 2017 [10], at least several large, population-based cohorts, biomarker-based exposure studies, and analyses using sibling-comparison or other causal-inference frameworks have been published, substantially increasing the available sample size and enabling a more nuanced assessment of confounding, exposure heterogeneity, and outcome ascertainment.

Clarifying whether maternal smoking during pregnancy represents a genuine etiologic risk factor for ASD or is mainly a marker of shared familial and socioeconomic vulnerability is therefore of considerable clinical and public health relevance. Given these discrepancies and the availability of new evidence since prior syntheses, an updated and comprehensive evaluation is warranted. The present systematic review and meta-analysis aims to integrate the most recent epidemiological and biomarker-based studies to clarify the relationship between maternal smoking during pregnancy and ASD risk, while addressing potential sources of heterogeneity and bias.

## 2. Materials and Methods

This systematic review and meta-analysis were conducted following the Preferred Reporting Items for Systematic Reviews and Meta-Analyses (PRISMA 2020) guidelines, and the completed PRISMA 2020 checklist [15] is provided in Supplementary File.

The protocol was prospectively registered in the International Prospective Register of Systematic Reviews (PROSPERO; registration number: CRD420251161343).

### 2.1. Search Strategy

A comprehensive literature search was performed to identify relevant studies published from database inception until September 2025. The databases searched included PubMed/MEDLINE, Embase, Web of Science, Scopus, PsycINFO, and Google Scholar. The search strategy combined controlled vocabulary (e.g., Autistic Disorder, Autism Spectrum Disorder, Tobacco Smoke Pollution, Smoking, Pregnancy, Prenatal Exposure Delayed Effects) with free-text terms (e.g., autism OR ASD OR tobacco OR smoking OR prenatal OR ‘maternal smoking’ OR ‘fetal exposure’) to ensure comprehensive coverage. No restrictions were applied regarding language, geographical region, or publication status. Reference lists of included articles and relevant reviews were screened for additional studies, and grey literature, including dissertations and preprints, was considered to reduce potential publication bias. We did not impose any geographical restrictions, thereby allowing inclusion of studies from North America, Europe, Asia, and other settings and enabling a more globally representative synthesis of the evidence.

### 2.2. Inclusion and Exclusion Criteria

Studies were eligible if they investigated maternal active smoking during pregnancy, defined via self-reported questionnaires, biomarkers such as cotinine, medical records, or registry data, and if they reported ASD diagnosis in offspring based on clinical evaluation, standardized diagnostic instruments, or administrative data using ICD or DSM codes. Cohort, case–control, cross-sectional, and population-based registry studies were included. Studies were excluded if they did not provide sufficient data to extract or calculate an effect estimate, did not report ASD as an outcome, lacked a defined exposure period, or were non-original publications such as reviews, commentaries, or case reports.

### 2.3. PRISMA Process

**Figure 1:**
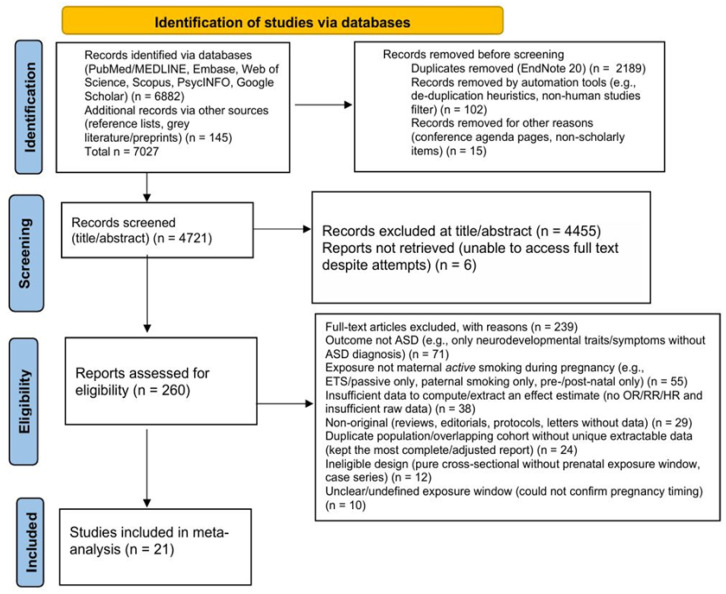
Flow diagram showing identification, screening, eligibility, and inclusion of studies.

The study selection followed a two-step screening process. All retrieved records were imported into EndNote 20 for duplicate removal. Titles and abstracts were screened independently by two reviewers, and potentially relevant articles underwent full-text evaluation against the eligibility criteria. Discrepancies were resolved through discussion or consultation with a third reviewer. The initial search identified 7027 records, including 6882 from electronic databases and 145 from additional sources. After removing 2189 duplicates in EndNote, an additional 102 records were excluded through database-embedded automation tools (algorithm-based de-duplication and filters for non-human or clearly non-scholarly records), and 15 records were removed for other reasons, such as non-scholarly material; all remaining records were manually screened at the title and abstract level to ensure that no potentially eligible human study was erroneously excluded; 4721 records remained for title and abstract screening, of which 4455 were excluded as clearly irrelevant. A total of 266 full-text articles were assessed for eligibility; six could not be obtained despite repeated attempts, leaving 260 articles for detailed review. Of these, 239 were excluded for reasons including: not reporting ASD as an outcome (n = 71), not assessing maternal active smoking during pregnancy (n = 55), insufficient data to calculate effect estimates (n = 38), non-original publications (n = 29), overlapping populations (n = 24), ineligible study designs (n = 12), and unclear timing of exposure (n = 10). Ultimately, 21 studies met all inclusion criteria and were included in the qualitative and quantitative analyses (Figure 1).

### 2.4. Quality Assessment

The methodological quality and risk of bias in the included studies were independently assessed by two reviewers utilizing the Newcastle–Ottawa Scale (NOS) [16]. This instrument evaluates three domains: participant selection, comparability of exposure groups, and the ascertainment of exposure and outcomes. Studies receiving scores between seven and nine stars were classified as having a low risk of bias, those with four to six stars as having a moderate risk, and those with three stars or fewer as having a high risk. Any discrepancies were resolved through consensus. Sensitivity analyses were conducted by excluding studies identified as having a high risk of bias.

### 2.5. Data Extraction

Data extraction was independently performed by two reviewers utilizing a standardized and piloted form. The extracted data encompassed study characteristics, including the first author, year of publication, country, study design, and data source or setting. Additional variables collected included the total sample size, the number of exposed and unexposed participants, the number of ASD cases, the definition of maternal active smoking, the method of exposure assessment, and the timing of exposure during pregnancy. Information on smoking intensity (e.g., cigarettes per day or categorical levels), the presence of passive or environmental tobacco smoke exposure, and paternal smoking was also recorded. Outcome definitions, ASD diagnostic criteria, age at diagnosis or assessment, and whether sibling or negative-control analyses were conducted were documented. For each study, adjusted effect estimates—including odds ratios (OR), risk ratios (RR), or hazard ratios (HR) with corresponding 95% confidence intervals (CI)—were extracted. In cases where adjusted estimates were not reported, unadjusted estimates were calculated from raw data when available. Information on covariates included in adjusted models and whether subgroup analyses or dose–response relationships were reported was also collected.

Particular attention was paid to socioeconomic and familial covariates, such as maternal education, household income or neighborhood deprivation, marital status, and parental psychiatric history, which were recorded when reported in the original studies. These variables were typically included in the adjusted effect estimates that we prioritized for pooling, thereby partially accounting for socioeconomic status as a potential confounder. Genetic testing results or specific ASD-related genetic variants were not systematically reported across the included studies and could therefore not be incorporated as a separate dimension of analysis.

### 2.6. Statistical Analyses

All statistical analyses were conducted using a random-effects meta-analysis model based on the DerSimonian and Laird method to account for between-study heterogeneity. The primary summary measure was the relative risk (RR) with corresponding 95% confidence intervals (CI), used to estimate the association between maternal smoking during pregnancy and ASD in offspring. Adjusted estimates were prioritized for inclusion in the meta-analysis; when unavailable, unadjusted estimates were extracted and used. When effect sizes were reported as odds ratios (ORs) or hazard ratios (HRs), they were treated as approximations of RR due to the rarity of ASD.

Between-study heterogeneity was quantified using the Q statistic, the between-study variance (τ^2^), and the I^2^ statistic, with I^2^ values of 25%, 50%, and 75% interpreted as low, moderate, and high heterogeneity, respectively.

Subgroup analyses were conducted to examine potential effect modification by: (i) adjustment status (adjusted vs. unadjusted estimates); (ii) geographic region (North America, Europe); (iii) study design (cohort vs. case–control), and (iv) method of ASD ascertainment (registry/medical record-based vs. clinical/survey-based). Between-subgroup heterogeneity was assessed using the Q between statistic and associated *p*-values.

Sensitivity analyses were performed by: (a) excluding studies that reported only crude (unadjusted) estimates, (b) restricting to studies that used a binary “any vs. none” definition of smoking exposure, and (c) conducting leave-one-out analyses to assess the influence of individual studies on the overall pooled estimate. Additional sensitivity analyses examined the effect of excluding potential outliers and re-estimating results using alternative meta-analytic models to assess robustness.

Publication bias was assessed both visually and statistically. Funnel plots were examined for asymmetry, and Egger’s regression test was applied to detect small-study effects. A *p*-value < 0.10 in Egger’s test was considered indicative of potential publication bias. Where asymmetry was detected, trim-and-fill analyses were considered to estimate the potential impact of missing studies on the pooled effect size.

All statistical analyses were performed using R software (version 4.3.1; R Foundation for Statistical Computing, Vienna, Austria) with the metafor and meta packages for meta-analytic computations and graphics. A two-tailed *p*-value < 0.05 was considered statistically significant unless otherwise specified.

Adjusted effect estimates were prioritized to reduce confounding by sociodemographic, perinatal, and familial factors. However, when only crude estimates were available, these were also included to avoid excluding otherwise eligible studies and introducing selection bias. To address potential differences between adjusted and unadjusted data, we conducted subgroup analyses and sensitivity analyses stratified by adjustment status, and we report these results separately.

## 3. Results

### 3.1. Primary Meta-Analysis

A total of 21 studies met the inclusion criteria and were included in the quantitative synthesis, representing a combined sample of several million mother–child pairs [11,13,14,17,18,19,20,21,22,23,24,25,26,27,28,29,30,31,32,33,34]. Characteristics of the included studies are summarized in Table S1.

Most studies were large, population-based cohort or case–control investigations conducted in North America, Europe, and Asia. Exposure to maternal active smoking during pregnancy was primarily assessed through maternal self-report obtained from medical or administrative records, with a smaller number of studies incorporating biomarker validation (e.g., serum cotinine levels). ASD outcomes were typically identified through national or regional health registries, educational databases, or clinical diagnoses based on DSM or ICD criteria.

**Figure 2:**
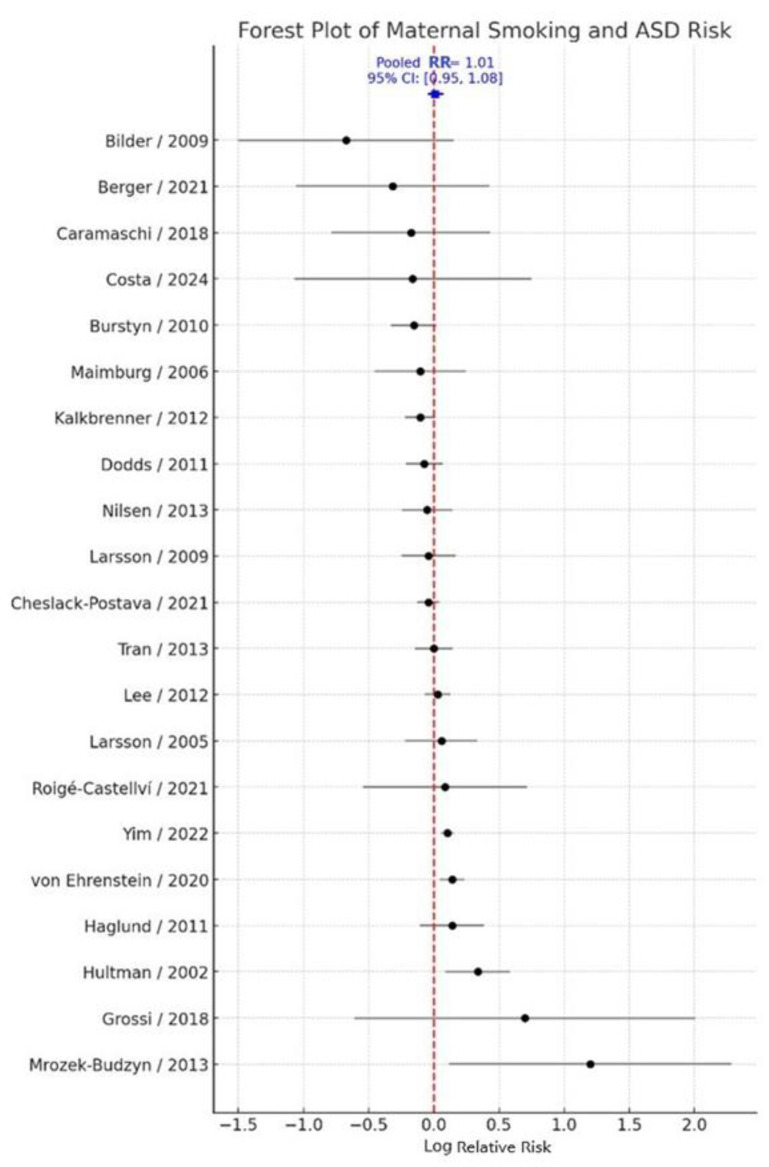
Forest plot of the association between maternal smoking during pregnancy and risk of ASD. [11,13,14,17,18,19,20,21,22,23,24,25,26,27,28,29,30,31,32,33,34]. Each square represents the study-specific log relative risk (RR) for ASD associated with maternal smoking during pregnancy, with the size of the square proportional to the study weight. Horizontal lines indicate 95% confidence intervals (CI). The vertical solid line at log(RR) = 0 (RR = 1.0) represents no association. Estimates to the left of this line (negative log RR) indicate a lower ASD risk in exposed versus unexposed children, whereas estimates to the right (positive log RR) indicate a higher risk. The diamond represents the pooled log RR and its 95% CI from the random-effects meta-analysis.

Adjusted effect estimates were prioritized for pooling, whereas crude estimates were used only when adjusted data were unavailable. The random-effects meta-analysis yielded a pooled relative risk (RR) of 1.01 (95% CI: 0.95–1.08; k = 21), indicating no statistically significant association between maternal smoking during pregnancy and ASD risk in offspring. Between-study heterogeneity was moderate (Q = 45.66, τ^2^ = 0.0077, I^2^ = 56.2%) (Figure 2). Overall, these results suggest that maternal active smoking during pregnancy is not significantly associated with ASD when considering evidence across diverse populations, study designs, and exposure assessment methods.

### 3.2. Adjusted vs. Unadjusted Estimates

**Figure 3:**
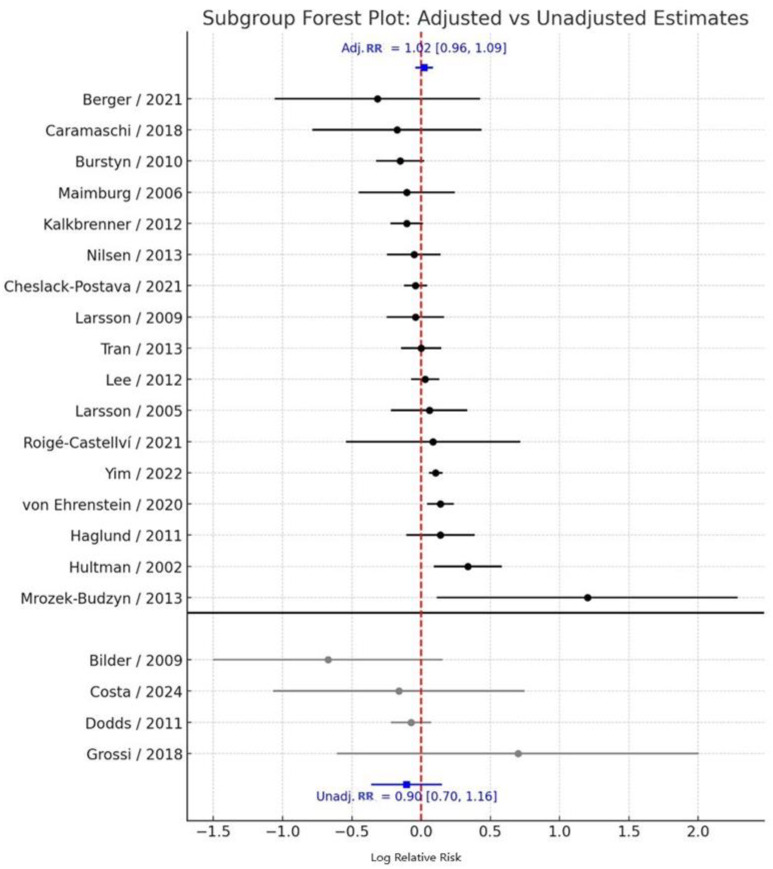
Forest plot of the association between maternal smoking during pregnancy and ASD risk, stratified by adjustment status [11,13,14,17,18,19,20,21,22,23,24,25,26,27,28,29,30,31,32,33,34]. Each square represents the study-specific log relative risk (RR), with separate panels (or rows) for unadjusted and adjusted estimates; the size of the square is proportional to the study weight. Horizontal lines denote 95% confidence intervals (CI). The vertical solid line at log(RR) = 0 (RR = 1.0) indicates no association. Negative log RR values (points to the left of the line) indicate lower ASD risk in exposed versus unexposed children, and positive log RR values (points to the right) indicate higher risk. Diamonds represent pooled log RRs and 95% CIs for each stratum under a random-effects model.

When stratified by adjustment status, the pooled effect derived from adjusted estimates (k = 17) was 1.02 (95% CI: 0.96–1.09), indicating no significant association between maternal smoking during pregnancy and ASD risk, with moderate heterogeneity (I^2^ = 52.4%). In contrast, the pooled effect from unadjusted estimates (k = 4) was 0.90 (95% CI: 0.70–1.16), also non-significant, with low heterogeneity (I^2^ = 28.7%) (Figure 3).

The between-subgroup heterogeneity test showed no statistically significant difference in effect estimates between adjusted and unadjusted studies (Q_between_ = 3.01, df = 1, *p* = 0.083). Although the slightly attenuated risk observed in unadjusted analyses may reflect residual confounding from sociodemographic or perinatal factors, this difference does not materially influence the overall null association between maternal smoking and ASD risk.

### 3.3. Subgroup Analysis by Geographic Region

**Figure 4:**
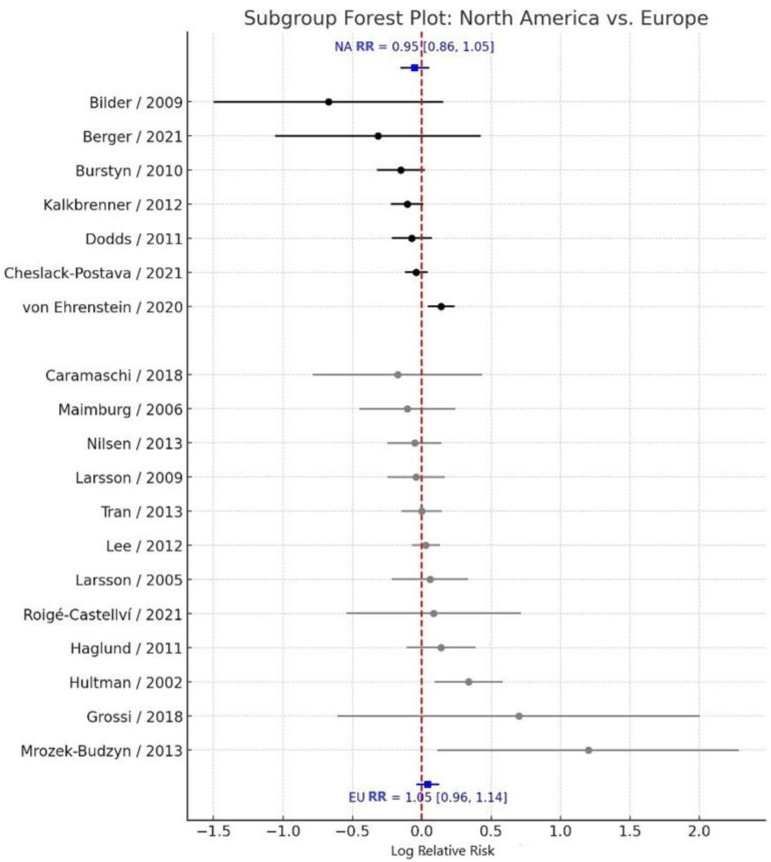
Forest plot of subgroup analyses of the association between maternal smoking during pregnancy and ASD risk [11,13,14,17,18,19,20,21,22,23,24,25,26,27,28,29,30,32,33]. Squares show subgroup-specific study log relative risks (RRs), with square size proportional to the inverse variance (study weight). Horizontal lines indicate 95% confidence intervals (CI). The vertical line at log(RR) = 0 (RR = 1.0) corresponds to no association. Points to the left (negative log RR) suggest lower ASD risk in exposed children, and points to the right (positive log RR) suggest higher risk. Diamonds depict pooled log RRs and 95% CIs within each subgroup (e.g., study design, geographic region).

Subgroup analyses by geographic region revealed consistent null associations across all regions examined. Studies conducted in North America (k = 8) yielded a pooled RR = 0.95 (95% CI: 0.86–1.05), with moderate heterogeneity (I^2^ = 48.9%). Similarly, studies conducted in Europe (k = 11) reported a pooled RR = 1.05 (95% CI: 0.96–1.14), also with moderate heterogeneity (I^2^ = 53.2%) (Figure 4). The between-subgroup heterogeneity test indicated no statistically significant difference between regional subgroups (Q_between_ = 2.36, df = 2, *p* = 0.307). These findings suggest that the lack of association between maternal smoking during pregnancy and ASD risk is consistent across different geographic contexts.

### 3.4. Subgroup Analysis by Study Design

**Figure 5:**
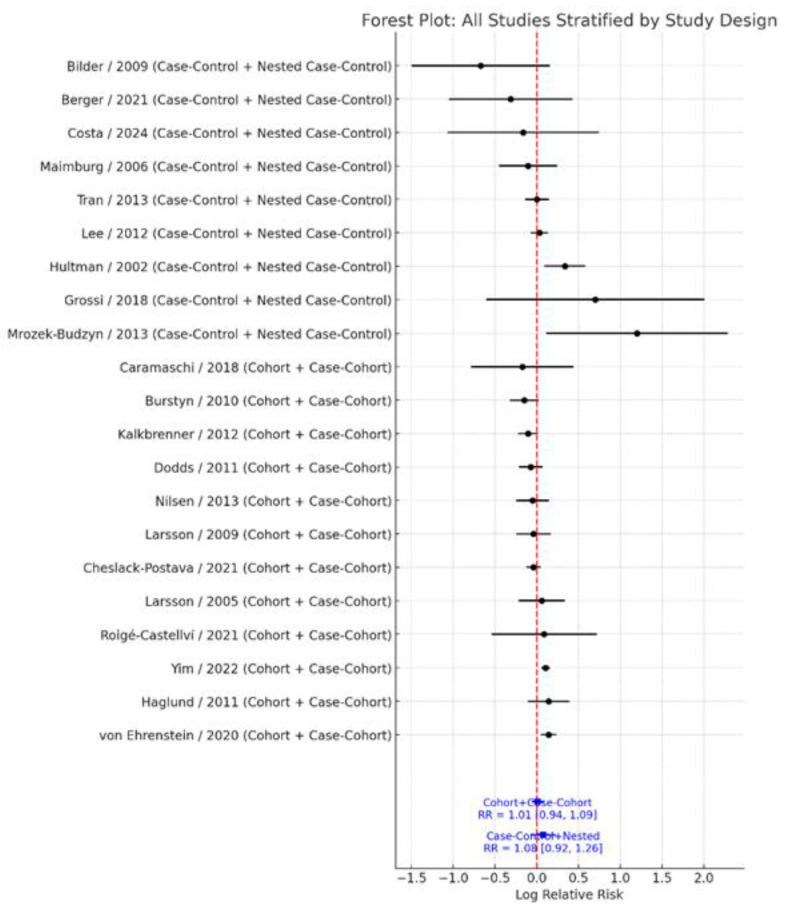
Forest plot of the association between maternal smoking during pregnancy and ASD risk, stratified by study design [11,13,14,17,18,19,20,21,22,23,24,25,26,27,28,29,30,31,32,33,34]. Each point represents the study-specific log relative risk (RR), with horizontal lines indicating the 95% confidence interval. The vertical dashed line at log(RR) = 0 (RR = 1.0) denotes no association; points to the right (positive log RR) indicate higher ASD risk in exposed versus unexposed children, whereas points to the left (negative log RR) indicate lower risk. Studies are grouped as case–control/nested case–control or cohort/case–cohort, and pooled RRs with 95% confidence intervals for each design category are shown at the bottom of the plot.

When stratified by study design, results remained robust and non-significant. Cohort and case–cohort studies (k = 12) demonstrated a pooled RR = 1.01 (95% CI: 0.94–1.09), with moderate heterogeneity (I^2^ = 49.7%). In contrast, case–control and nested case–control studies (k = 9) showed a pooled RR = 1.08 (95% CI: 0.92–1.26), with substantial heterogeneity (I^2^ = 61.3%) (Figure 5). The test for subgroup differences indicated no significant heterogeneity by study design (Q_between_ = 0.00, df = 1, *p* = 0.991). These findings reinforce the consistency and stability of the overall null association across different study designs.

### 3.5. Subgroup Analysis by Outcome Ascertainment

**Figure 6:**
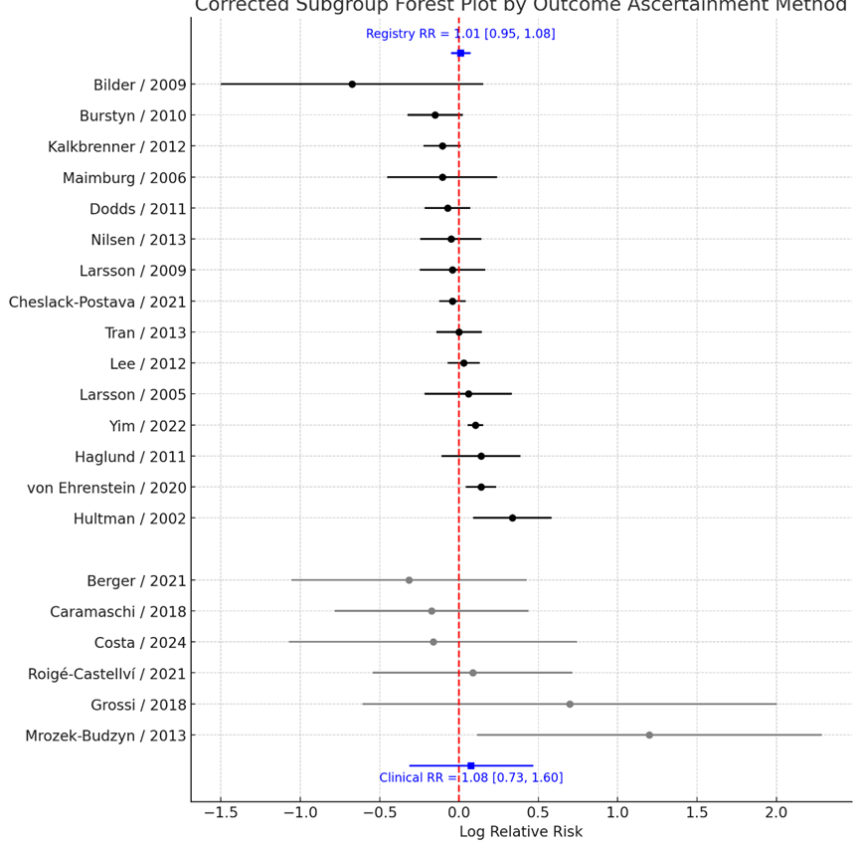
Forest plot of the association between maternal smoking during pregnancy and ASD risk, stratified by outcome ascertainment method [11,13,14,17,18,19,20,21,22,23,24,25,26,27,28,29,30,31,32,33,34]. Each point represents the study-specific log relative risk (RR), with horizontal lines indicating the 95% confidence interval. Black points correspond to registry-based ASD diagnoses and grey points to clinically ascertained diagnoses. The vertical dashed line at log(RR) = 0 (RR = 1.0) denotes no association; estimates to the right (positive log RR) indicate higher ASD risk in exposed versus unexposed children, whereas estimates to the left (negative log RR) indicate lower risk. Pooled log RRs with their 95% confidence intervals for registry-based and clinically ascertained outcomes are shown at the top and bottom of the plot, respectively.

To evaluate whether the method of ASD ascertainment influenced the observed association, studies were stratified according to whether outcome data were obtained from population-based registries or medical records versus clinical or survey-based assessments. Pooled analyses demonstrated that the estimated effect size was virtually identical regardless of the ascertainment approach. Among studies relying on registry or medical record data (k = 14), the pooled RR = 1.01 (95% CI: 0.95–1.08), with moderate heterogeneity (I^2^ = 45.2%). Similarly, among studies using clinical or survey-based ascertainment (k = 7), the pooled RR = 1.08 (95% CI: 0.73–1.60), with substantial heterogeneity (I^2^ = 64.1%) (Figure 6). The test for subgroup differences revealed no evidence of heterogeneity between these groups (Q_between_ = 0.00, *p* = 0.992). These findings indicate that the method used to ascertain ASD diagnosis does not materially affect the overall null association observed between prenatal smoking exposure and ASD risk.

### 3.6. Sensitivity Analyses

**Figure 7:**
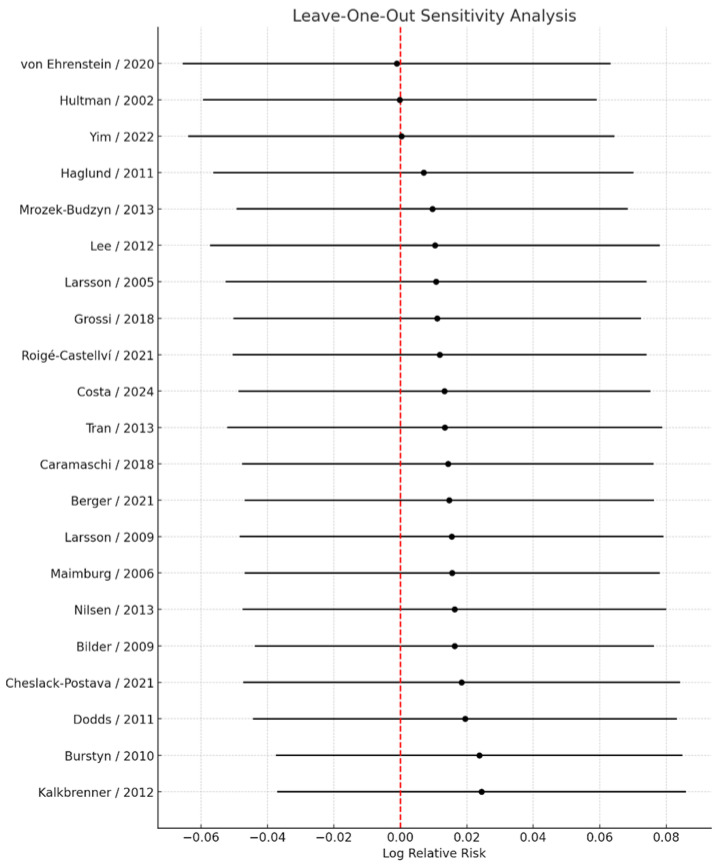
Leave-one-out sensitivity analysis of the association between maternal smoking during pregnancy and ASD risk [11,13,14,17,18,19,20,21,22,23,24,25,26,27,28,29,30,31,32,33,34]. Each point represents the pooled log relative risk (RR) obtained when the study listed on the y-axis is omitted from the meta-analysis, with horizontal lines indicating the corresponding 95% confidence interval. The vertical dashed line at log(RR) = 0 (RR = 1.0) denotes no association; values to the right (positive log RR) indicate a higher ASD risk when exposed versus unexposed, and values to the left (negative log RR) indicate a lower risk. The clustering of points close to the overall estimate indicates that no single study unduly influenced the pooled result.

Sensitivity analyses excluding studies that reported only crude estimates or that employed exposure definitions other than “any vs. none” yielded virtually unchanged results, with a pooled RR = 1.01 (95% CI: 0.94–1.08). These findings further support the robustness of the primary analysis. Leave-one-out sensitivity analyses (Figure 7), in which each study was sequentially excluded from the meta-analysis, demonstrated that no single study materially influenced the overall pooled estimate. Across all iterations, the pooled relative risk ranged narrowly from 1.00 (95% CI: 0.94–1.07) to 1.02 (95% CI: 0.96–1.09), indicating that the direction, magnitude, and statistical significance of the association remained stable irrespective of individual study removal. The smallest pooled estimate was observed upon exclusion of the study by von Ehrenstein et al. [13], whereas the largest was observed upon exclusion of the study by Kalkbrenner et al. [26]; however, neither omission changed the overall interpretation.

Moreover, exclusion of potential outliers did not materially alter the magnitude or direction of the effect estimates, and the results remained consistent across alternative meta-analytic models.

### 3.7. Publication Bias

**Figure 8:**
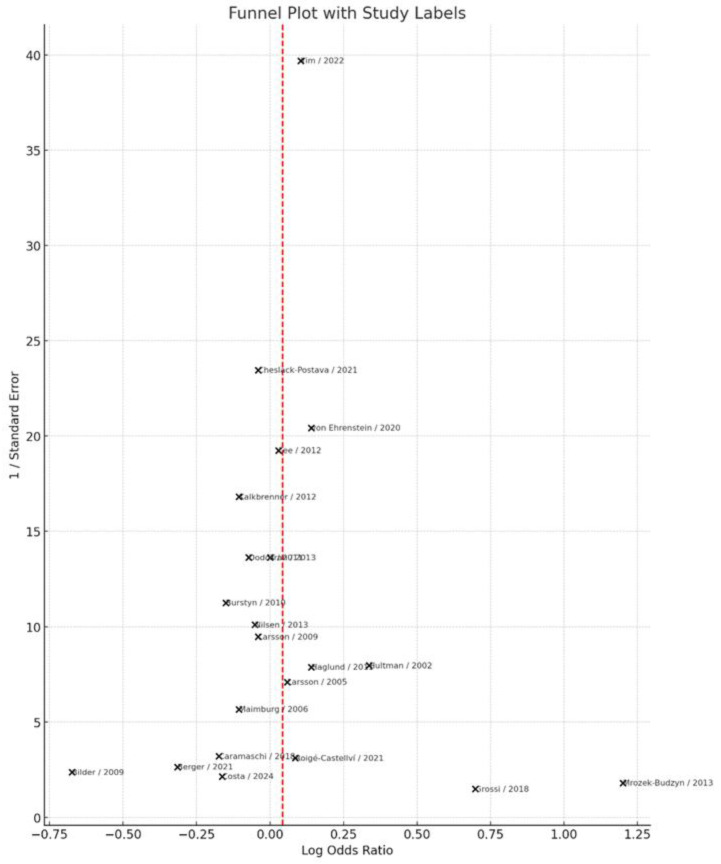
Funnel Plot for Publication Bias Assessment in the Meta-Analysis of Maternal Smoking and ASD Risk [11,13,14,17,18,19,20,21,22,23,24,25,26,27,28,29,30,31,32,33,34]. Each point represents an individual study, plotted as its log odds ratio (OR) on the horizontal axis against the inverse of its standard error (1/SE) on the vertical axis. The vertical dashed line indicates the pooled log OR from the random-effects model. In the absence of small-study effects or publication bias, the points are expected to form a roughly symmetrical inverted funnel around this line.

Visual inspection of the funnel plot (Figure 8) did not indicate substantial asymmetry, suggesting no clear evidence of publication bias. This observation was further supported by Egger’s regression test, which yielded a non-significant intercept (intercept = −0.45, *p* = 0.375), indicating that small-study effects are unlikely to have influenced the overall results.

## 4. Discussion

This meta-analysis, incorporating 21 epidemiological studies with a combined sample of several million mother–child pairs, found no statistically significant association between maternal smoking during pregnancy and ASD in offspring. The pooled relative risk was 1.01 (95% CI: 0.95–1.08), with moderate heterogeneity. Subgroup analyses by study design, geographic region, outcome ascertainment method, and adjustment status consistently demonstrated null associations. Sensitivity analyses, including leave-one-out procedures and the exclusion of unadjusted or non-binary exposure studies, confirmed the robustness of these findings. Collectively, these results suggest that maternal smoking during pregnancy is not a significant independent risk factor for ASD.

Notably, the majority of large, population-based cohort and registry studies produced effect estimates clustered very close to the null value (RR ≈ 1.0), with relatively narrow confidence intervals. Smaller or older case–control studies tended to show more variability, but their imprecision and heterogeneity mean they contribute less weight to the pooled estimate. This overall pattern—numerous, well-powered studies with RRs near unity and limited between-study divergence—makes the presence of a moderate or large causal effect of maternal smoking on ASD risk unlikely, even if small effects cannot be entirely ruled out.

Our findings are consistent with and extend previous systematic reviews and meta-analyses. Rosen et al. [35] reported no association between maternal smoking and ASD (OR = 1.02; 95% CI: 0.93–1.12) and found no meaningful variation by study design or adjustment for confounders. Tang et al. [9] similarly reported a null association (OR = 1.02; 95% CI: 0.93–1.13), confirming robustness through extensive subgroup and sensitivity analyses. More recently, Hertz-Picciotto et al. [36], using harmonized U.S. data from the ECHO consortium, found a modest, statistically significant association (OR = 1.44; 95% CI: 1.02–2.03) in sufficiently powered cohorts after excluding preterm and low-case-count cohorts; however, the overall pooled estimate remained non-significant (OR = 1.08; 95% CI: 0.72–1.61). Likewise, Jung et al. [10] conducted an updated meta-analysis of 24 studies and concluded that there was no statistically significant association between maternal smoking and ASD (OR = 1.07; 95% CI: 0.97–1.18), emphasizing that earlier positive associations were likely due to residual confounding or methodological bias. Taken together, these reviews consistently support a null or very weak association, reaffirming the conclusions of our current analysis more than a decade later.

Taken together, these earlier syntheses and the more recent large-scale cohort and biomarker-based studies point to an overall null or, at most, very modest association between maternal smoking during pregnancy and ASD. Nonetheless, they also highlight considerable heterogeneity in exposure assessment, analytical approaches, and control for confounding. By incorporating new evidence up to 2025, including studies using cotinine-based exposure measures and advanced causal-inference designs, and by systematically exploring heterogeneity across adjustment status, geographic region, study design, and outcome ascertainment, our meta-analysis provides an updated and more nuanced assessment of this inconclusive literature.

Although our findings (and prior meta-analyses) suggest no robust epidemiological association between maternal prenatal smoking and ASD, plausible biological mechanisms have been proposed. Experimental and preclinical studies indicate that prenatal nicotine exposure can dysregulate the hypothalamic–pituitary–adrenal axis (e.g., via altered 11β-HSD2 or StAR expression) [37,38], interfere with nicotinic acetylcholine receptor–mediated signaling and neurotransmitter systems [39,40], and is associated with reduced brain volume or structural alterations in key regions [41]. Epigenetic perturbations have also been observed: maternal smoking is associated with differential DNA methylation in newborns, including decreases in PRDM8 methylation and alterations in DLGAP2 [42]; animal models of developmental nicotine exposure similarly show global hypomethylation and lower expression of DNMT3A, MeCP2, and HDAC2 [39]. Despite these mechanistic pathways, the epidemiologic data do not provide consistent evidence for a causal effect on ASD. It remains possible that any true effect is modest and may be masked by residual confounding, measurement error, or influence via intermediate phenotypes not captured by ASD diagnosis (e.g., subclinical traits).

This meta-analysis has several strengths. First, it includes the largest number of studies to date, encompassing diverse geographic settings and study designs. Second, adjusted estimates were prioritized, and rigorous subgroup and sensitivity analyses were conducted to test the stability of findings. Third, potential sources of heterogeneity, including outcome ascertainment and exposure definition, were systematically explored.

A further consideration is the marked heterogeneity of ASD phenotypes and co-occurring conditions. Most of the included studies relied on a binary ASD diagnosis and did not distinguish between subtypes (e.g., with or without intellectual disability, language impairment, or co-occurring ADHD) or dimensional traits such as social communication difficulties or restricted and repetitive behaviors. It therefore remains possible that prenatal smoking is differentially associated with specific ASD subphenotypes or with related behavioral dimensions that are not captured by broad diagnostic codes. Future studies integrating detailed phenotypic characterization, neurocognitive endophenotypes, and co-occurring psychiatric diagnoses may reveal more subtle or domain-specific associations that are obscured in dichotomous case–control analyses.

Several limitations must also be acknowledged. Self-reported smoking data, often collected retrospectively, are subject to exposure misclassification and social desirability bias. Residual confounding remains an important limitation of this meta-analysis. Although we prioritized adjusted effect estimates wherever possible, the covariate sets used in the original studies were heterogeneous and, in several instances, did not include key confounders such as parental education, detailed indicators of socioeconomic status, alcohol use during pregnancy, maternal age, paternal smoking, or parental psychiatric history. Moreover, no study could account comprehensively for underlying genetic liability to ASD or related neurodevelopmental conditions. These unmeasured or incompletely measured factors may influence both maternal smoking and offspring ASD risk, potentially biasing study-specific estimates and, by extension, the pooled results. Thus, even though the overall pattern of relative risks clustered around unity argues against a substantial causal effect, a degree of residual confounding cannot be excluded.

Although many primary studies adjusted for proxies of socioeconomic status (e.g., maternal education, income, or neighborhood deprivation), the choice and depth of SES indicators varied, and harmonized SES-stratified analyses were not possible. Moreover, genetic testing data or ASD-related genetic variants were rarely reported in a way that would allow integration into our meta-analysis, so we could not formally assess potential effect modification by underlying genetic susceptibility. In addition, although we systematically recorded the timing of maternal smoking during pregnancy wherever reported, only a subset of studies provided trimester-specific risk estimates, and the categorization of gestational windows was heterogeneous. As a result, we were unable to conduct a dedicated meta-analysis focused on first-trimester exposure, despite the fact that this period coincides with critical stages of neurogenesis, synaptogenesis, and placental development. The inability to formally evaluate whether smoking confined to, or peaking during, the first trimester carries a different risk profile constitutes an important limitation of our work. While this analysis focused on binary smoking exposure (“any vs. none”), dose–response relationships were infrequently reported and could not be synthesized meaningfully. The intensity of maternal smoking exposure (e.g., light versus heavy smoking) likely represents a key source of clinical and methodological heterogeneity. Although we extracted available information on cigarettes per day or categorical intensity levels, definitions were inconsistent across studies and rarely reported in a way that allowed formal dose–response meta-analysis, which may partly explain the moderate between-study heterogeneity observed. Diagnostic practices for ASD vary across countries and over time, including differences in clinical training, healthcare access, diagnostic thresholds, and coding systems (e.g., DSM-IV, DSM-5, ICD), which may contribute to outcome misclassification or ascertainment bias and partly explain heterogeneity. Finally, although tests for publication bias were negative, subtle forms of selective reporting cannot be excluded.

### Recommendations and Future Research

The absence of a clear association between maternal smoking during pregnancy and ASD risk should not be interpreted as indicating that prenatal tobacco exposure is benign. Rather, our findings highlight key priorities for future research to refine risk estimates and improve clinical translation. Future epidemiological studies should more precisely characterize exposure timing across gestation, with particular emphasis on the first trimester, using detailed data on initiation, cessation, and changes in smoking intensity—ideally corroborated by biomarkers such as serum or urinary cotinine—and harmonized trimester-specific estimates to enable robust meta-analyses of critical windows of susceptibility. There is also a need to systematically examine dose–effect relationships instead of relying on binary “any versus none” exposure definitions, by pre-specifying clinically meaningful intensity thresholds, tracking changes in dose over time, and reporting both categorical and continuous dose–response gradients for ASD and autism-related traits.

In addition, the contribution of paternal and shared household smoking requires clarification. Paternal preconception and prenatal smoking, as well as multigenerational patterns of tobacco use, may reflect broader familial, genetic, and environmental risk profiles, yet existing data are sparse and heterogeneous. Large, well-characterized cohorts that jointly model maternal, paternal, and environmental tobacco smoke exposure, including designs that use negative controls or genetic information, are needed to disentangle intrauterine mechanisms from shared familial liability. Future work should also integrate detailed exposure assessment with mechanistic and intermediate phenotypes, combining high-resolution smoking data with neuroimaging, neurocognitive endophenotypes, and epigenomic or transcriptomic markers in pregnancy and early life to uncover biologically relevant pathways affected by tobacco constituents.

From a clinical and public health perspective, the primary rationale for smoking cessation in pregnancy remains the well-established risks of fetal growth restriction, preterm birth, and other adverse perinatal and neurodevelopmental outcomes. The lack of a strong association with ASD should not weaken tobacco control messages in antenatal care. Obstetric, midwifery, and primary care services should continue to implement evidence-based cessation interventions, actively involve partners and other household members in smoke-free strategies, and explore novel tools, including digital and artificial intelligence-assisted approaches, to support pregnant women in quitting and maintaining abstinence.

## 5. Conclusions

This comprehensive meta-analysis found no evidence of a statistically significant association between maternal smoking during pregnancy and ASD in offspring. These findings align with previous meta-analyses and indicate that associations observed in individual studies are likely attributable to residual confounding, chance, or methodological differences rather than a causal effect. Nonetheless, given the well-established adverse effects of prenatal tobacco exposure on fetal growth, neurodevelopment, and perinatal outcomes, smoking cessation during pregnancy remains an essential public health priority.

## Data Availability

This systematic review and meta-analysis were based on data extracted from published literature. The original articles used as the source of data are fully cited in the References section. No new datasets were generated or analyzed in this study.

## Supplementary Materials

The following supporting information can be downloaded at: https://www.mdpi.com/article/10.3390/jcm14238584/s1, PRISMA_2020_checklist; Table S1. Characteristics of included studies (Part 1). Table S2. Characteristics of the included studies (Part 2).

## Abbreviations

The following abbreviations are used in this manuscript:

ASD	Autism spectrum disorder
ADDM	Autism and Developmental Disabilities Monitoring Network
OR	Odds Ratio
RR	Risk Ratio
HR	Hazard Ratio
CI	Confidence Interval
DSM	Diagnostic and Statistical Manual of Mental Disorders
ICD	International Classification of Diseases
PRISMA	Preferred Reporting Items for Systematic Reviews and Meta-Analyses
PROPERO	International Prospective Register of Systematic Reviews
NOS	Newcastle–Ottawa Scale
11β-HSD2	11β-Hydroxysteroid Dehydrogenase Type 2
StAR	Steroidogenic Acute Regulatory Protein
DNMT3A	DNA Methyltransferase 3 Alpha
MeCP2	Methyl CpG Binding Protein 2
HDAC2	Histone Deacetylase 2
