# Supplementary material for: Maternal Smoking During Pregnancy and Risk of Autism Spectrum Disorder in Offspring: A Systematic Review and Meta-Analysis
Source: J Clin Med. 2025 Dec 3;14(23):8584. doi: 10.3390/jcm14238584 (PMC12692760; doi:10.3390/jcm14238584)
Supplement: Supplementary file 1 [file jcm-14-08584-s001.zip › jcm-3988674-supplementary.pdf]

SUPPLEMENTARY FILE

Table S1. Characteristics of the included studies (Part 1).

First author/ Year	Country	Design	Setting/Data source	Population/Eligibility	Sample size (total)	Exposed (n)	Unexposed (n)	ASD cases (n)	Exposure definition (maternal active smoking)	Exposure assessment method	Timing of exposure	Smoking intensity categories
Bilder / 2009 [17]	USA	Population-based nested case–control	Utah ADDM Network – linked medical, educational, and developmental disability service records	All children born in 1994, residing in the 3 most populous Utah counties and aged 8 in 2002	26,315	ASD: 6; Controls: 1,244	ASD: 114; Controls: 11,956	120	Maternal cigarette smoking during pregnancy (any use)	Birth certificate (maternal self-report)	Entire pregnancy (not trimester-specific)	Binary (any vs none)
Burstyn / 2010 [18]	Canada	Population-based retrospective cohort	Alberta Perinatal Health Program (APHP) linked with Alberta Health and Wellness registries	Singleton live births in Alberta (1998–2004) with linked maternal and child health data	218,890	45,846	169,374	1,138 (≥1 ASD claim)	Maternal self-reported smoking during pregnancy (any use)	Maternal self-report in APHP database at delivery admission	Entire pregnancy (no trimester-specific breakdown)	Binary (any vs none)

Dodds / 2011 [19]	Canada	Population-based retrospective cohort	Nova Scotia Atlee Perinatal Database linked with provincial physician billing, hospital discharge, and mental health records	All singleton live births in Nova Scotia (1990–2002) with linked maternal and child data and follow-up for ASD	129,733	≈20,300 (~15.7%)	≈109,000 (~84.3%)	924	Maternal self-reported smoking during pregnancy (any use)	Maternal self-report from perinatal database	Entire pregnancy (not trimester-specific)	Binary (any vs none)
Haglund / 2011 [20]	Norway	Population-based cohort	Norwegian Mother, Father and Child Cohort Study (MoBa) linked with Norwegian Patient Registry	Singleton live births enrolled in MoBa (1999–2009) with linked maternal questionnaire data and ASD outcomes	104,828	~8,260 (~7.9%)	~96,568 (~92.1%)	617	Maternal self-reported smoking during pregnancy (any smoking)	Maternal questionnaire data during pregnancy	Entire pregnancy (no trimester-specific breakdown)	Binary (any vs none)
Hultman / 2002 [21]	Sweden	Population-based nested case–control	Swedish Medical Birth Register linked with National	All singleton live births in Sweden (1974–1993) with linked maternal data and	≈1,465,000	NR	NR	408	Maternal daily smoking during pregnancy	Antenatal care record (self-report by midwives)	Early pregnancy (first prenatal visit,	Binary (daily vs nondaily)

			Inpatient Register	inpatient ASD diagnoses					(daily vs nondaily)		typically <15 weeks)	
Larsson / 2005 [22]	Sweden	Population-based cohort	Swedish Medical Birth Register linked with National Hospital Discharge and Outpatient Registers	All singleton live births in Sweden (1987–1999) with linked maternal smoking data and ASD diagnoses	1,418,292	≈247,000 (~17.4%)	≈1,171,000 (~82.6%)	4,055	Maternal smoking during pregnancy (any smoking) reported at first antenatal visit	Midwife-recorded maternal self-report at first antenatal care visit	Early pregnancy (first antenatal visit, typically first trimester)	Binary (any vs none)
Larsson / 2009 [23]	Sweden	Population-based cohort	Swedish Medical Birth Register linked with National Patient Register	All singleton live births in Sweden (1987–2001) with maternal smoking data and follow-up for ASD diagnosis	1,209,346	≈205,200 (~17.0%)	≈1,004,000 (~83.0%)	3,958	Maternal smoking during pregnancy (any vs none, categories: none, 1–9/day, ≥10/day)	Midwife-recorded maternal self-report during routine prenatal care	Early pregnancy (first antenatal visit, typically first trimester)	Binary (any vs none); also categorical (none, light, heavy)
Kalkbrenner / 2012 [24]	USA	Population-based case–cohort	Autism and Developmental Disabilities Monitoring (ADDM) Network	All singleton live births in 11 U.S. states (1992, 1994, 1996, 1998) with ASD case ascertainment	633,989	≈13% overall maternal smoking (~11% among	≈87% non-smoking	3,315	Maternal self-reported smoking during pregnancy (any use)	Birth certificate records	Entire pregnancy (not trimester-specific)	Binary (any vs none)

			linked with state birth certificate records	through ADDM surveillance		ASD cases)						
Lee / 2012 [25]	Sweden	Population-based nested case–control	Stockholm Youth Cohort linked with Swedish national and regional registers	All children born 1984–2003, residing in Stockholm County ≥4 years, with ASD diagnosis and maternal smoking data	42,941 (3,958 ASD cases, 38,983 controls)	783 ASD cases (~19.8%)	3,175 ASD cases (~80.2%)	3,958	Maternal smoking during pregnancy (none / 1–9 / ≥10 cigarettes daily)	Maternal self-report at first prenatal visit (~8–12 weeks) in the Medical Birth Register	Early pregnancy (first antenatal visit)	Categorical (none, 1–9/day, ≥10/day)
Maimburg / 2006 [26]	Denmark	Population-based case–control	Danish Medical Birth Register linked with Danish Psychiatric Central Register	All singleton live births in Denmark (1990–1999) with linked maternal smoking data and infantile autism diagnoses	5,203 (473 cases, 4,730 matched controls)	111 cases (~29%), 1,049 controls (~28%)	362 cases (~71%), 3,681 controls (~72%)	473	Maternal smoking during pregnancy (any use) recorded at first antenatal visit	Midwife-recorded maternal self-report during antenatal care	Early pregnancy (first antenatal visit, ~12 weeks)	Binary (any vs none)
Nilsen / 2013 [27]	Norway	Population-based cohort	Norwegian Mother and Child Cohort Study (MoBa) linked with Norwegian	Singleton live births in MoBa (1999–2008) with maternal smoking data and ASD follow-up	86,700	≈6,200 (~7.2%)	≈80,500 (~92.8%)	376	Maternal self-reported smoking during pregnancy (none, occasional,	Maternal questionnaire data collected during pregnancy	Weeks 17 and 30 of pregnancy	Binary (any vs none); also occasional vs daily categories

			Patient Registry						daily – combined as any vs none in main analysis)			
Mrozek-Budzyn / 2013 [28]	Poland	Case–control	Regional psychiatric outpatient clinic and general practice controls in Malopolska Voivodship	Children aged 2–15 years diagnosed with ASD and age- and sex-matched controls	288 (96 cases; 192 controls)	12 cases (~12.5%)	84 cases (~87.5%)	96	Maternal active smoking during pregnancy (any use)	Maternal structured interview questionnaire (retrospective)	Entire pregnancy (retrospectively reported)	Binary (any vs none)
Tran / 2013 [29]	Finland	Population-based nested case–control	Finnish Medical Birth Register linked with Finnish Hospital Discharge Register (FIPS-A study)	All live births in Finland (Oct 1990–Dec 2005) with linked maternal smoking data and ASD outcomes	≈20,142 (4,019 ASD cases, 16,123 matched controls)	≈16.0% continued smoking throughout pregnancy; ≈1.9% quit after first trimester	≈82.1% non-smokers	4,019	Maternal smoking during pregnancy categorized as: none, quit after first trimester, or continued throughout pregnancy	Maternal self-report on perinatal data collection forms (recorded by nurses)	First trimester only vs continued throughout pregnancy	Categorical (none / quit after first trimester / continued throughout pregnancy)

Berger/2021 [30]	USA	Case–control	California Prenatal Screening Program, linked with DDS and birth records	Singleton children with stored maternal serum samples in 2nd trimester, cases with ASD in DDS database, controls without DDS linkage	997 (498 cases, 499 controls)	16 (cotinine ≥3.08 ng/ml)	981	498	Maternal active smoking defined by cotinine ≥3.08 ng/ml or self-report of smoking during pregnancy	Biomarker (serum cotinine) and self-report from birth records	Second trimester (~15–20 weeks gestation)	Light ≤35 ng/ml vs heavy >35 ng/ml (cotinine-based)
Caramaschi/2018 [11]	UK	Birth cohort	Avon Longitudinal Study of Parents and Children (ALSPAC)	Singleton children from ALSPAC with data on maternal smoking and ASD outcomes	≤12,044 (maternal smoking sample)	~3700 (~30.7%)	~8300	212 (174 with pregnancy data)	Any maternal smoking during pregnancy (self-report) and methylation score proxy	Self-report (questionnaires at 8, 18–32 weeks, postnatal); DNA methylation score; CHRNA3 genotype (MR)	Across pregnancy and by trimester	Continuous (cigarettes/day); also categorical (any vs none)
Costa / 2024 [31]	Brazil	Case–control	ASD outpatient clinic (cases) and primary care controls	Children/adolescents with ASD and neurotypical controls	1,134 (248 cases; 886 controls)	31 (6 cases; 25 controls)	1,103	248	Maternal smoking during pregnancy (self-report)	Structured maternal interview	Entire pregnancy (no trimester breakdown)	Not reported

Grossi / 2018 [32]	Italy	Case–control	Two autism centers (Villa Santa Maria & Stella Maris)	Mothers of children with ASD, siblings, and typically developing controls	249 pregnancies (73 ASD, 45 siblings, 96 controls)	8.33% of ASD mothers	4.35% of controls	73	Smoking during pregnancy defined as ≥5 cigarettes/day (self-report)	Structured maternal interview (face-to-face)	Conception and entire pregnancy	Not reported (binary yes/no)
Cheslack-Postava/2021 [14]	USA	Retrospective cohort	Massachusetts birth certificate registry	Singleton live births in Massachusetts with linked special education ASD classification	653,913	11.4%	88.6%	3,957	Maternal self-reported smoking during pregnancy on birth certificate	Birth certificate	Entire pregnancy (not trimester-specific)	Binary (any vs none)
Roigé-Castellví/2021 [33]	Spain	Population-based cohort	INMA Project (Spanish Birth Cohort)	Singleton children with maternal smoking data and neurodevelopmental follow-up	2,139	355 (16.6%)	1,784	57	Any maternal smoking during pregnancy (self-report)	Self-administered questionnaire at 12 and 32 weeks gestation	Entire pregnancy and trimester-specific	Categorical: none, ≤10, >10 cigarettes/day
von Ehrenstein / 2020 [13]	USA	Population-based cohort	California Birth Statistical Master Files linked with California Department	All singleton live births in California (2007–2010) with ASD ascertainment from DDS records	2,015,104	~48,110 (~2.4%)	~1,923,831 (~96%)	11,722	Maternal self-reported smoking during pregnancy recorded	Birth certificate	Entire pregnancy (per trimester also recorded)	Continuous (cigarettes/day); categorical (1–19

			of Developmental Services (DDS)						on birth certificate			vs ≥20/day)
Yim/2022 [34]	South Korea	Nationwide retrospective cohort	Korean National Health Insurance Service (NHIS) database	Singleton live births with linked maternal and child health data	2,444,904	30,512 (1.25%)	~2,414,392	12,390	Maternal self-reported smoking during pregnancy (perinatal visit)	Self-reported via prenatal care questionnaire	Entire pregnancy (no trimester-specific detail)	Binary (any vs none)

Table S2. Characteristics of the included studies (Part 2).

First author/ Year	Passive/environmental tobacco smoke assessed?	Paternal smoking assessed?	Negative-control or sibling analyses	Outcome definition	Outcome ascertainment	Age at ASD diagnosis/assessment	Effect type	Main adjusted estimate (value; 95% CI)	Comparison encoded	Adjustment set	Subgroup estimates reported	Dose–response reported	NOS total stars

Bilder / 2009 [17]	No	No	No	DSM-IV-TR	Multi-source abstraction (medical, education, special education records) reviewed and coded by CDC-trained abstractors	8 years	Odds ratio (unadjusted)	0.51 (95% CI: 0.22–1.15)	Ever vs never	Not adjusted for this exposure; perinatal/neonatal models adjusted for maternal age, gestational age, parity	No	No	8
Burstyn / 2010 [18]	No	No	No	ICD-9 299.0, 299.8 autism spectrum disorder diagnosis	Alberta Health and Wellness administrati	Follow-up until 31 March 2008 (peak ASD diagnosis	Adjusted Relative Risk (log-binomial	0.86 (95% CI: 0.72–1.02)	Any smoking vs none	Adjusted for maternal age, parity, socioeconomic status, gestational age, infant sex, maternal	No	No	8

					ve records	~3–4 years)	regression)			anthropometrics, diabetes, gestational complications, birth year			
Dodds / 2011 [19]	No	No	No	ICD-9 299 / ICD-10 F84 autism spectrum disorder diagnosis	Linked provincial physician billing, hospital discharge, and outpatient mental health databases	1–17 years (peak diagnosis ~3 years)	Relative risk (unadjusted)	0.93 (95% CI: 0.81–1.08)	Ever vs never	Not included in final adjusted model; final model adjusted for birth year, income support, pre-pregnancy weight, maternal medical conditions, interpregnancy interval, parity, weight gain, type of labour, infant sex, breastfeeding, and CNS anomaly			

Haglund / 2011 [20]	No	Yes – paternal smoking reported	No	ICD-10 F84 autism spectrum disorder diagnosis	Norwegian Patient Registry diagnostic records linked to cohort data	Median follow-up ~8.2 years	Adjusted odds ratio (aOR)	1.15 (95% CI: 0.90–1.48)	Ever vs never	Maternal age, parity, parental education, income, parental psychiatric history, birth year			
Hultman / 2002 [21]	No	No	No	ICD-9 299A autism diagnosis	National Inpatient Register hospitalization records	Diagnosis before age 10	Adjusted odds ratio (conditional logistic regression)	1.4 (95% CI: 1.1–1.8)	Daily vs nondaily	Adjusted for maternal age, parity, maternal country of birth, pregnancy bleeding, hypertensive disease, diabetes, mode of delivery, gestational age, size for gestational			

										age, Apgar score, congenital malformations			
Larsson / 2005 [22]	No	No	No	ICD-9 299 / ICD-10 F84 autism spectrum disorder diagnosis	National inpatient and outpatient health registers	Diagnosis before age 10	Adjusted relative risk (RR)	1.06 (95% CI: 0.80–1.39)	Ever vs never	Adjusted for maternal age, parity, parental psychiatric history, socioeconomic status	No	No	8
Larsson / 2009 [23]	No	No	No	ICD-9 299 / ICD-10 F84 autism spectrum disorder diagnosis	National Patient Register (inpatient and outpatient care records)	Follow-up to 2006 (median diagnosis age ≈ 6.2 years)	Adjusted relative risk (aRR, Poisson regression)	0.96 (95% CI: 0.78–1.18)	Ever vs never	Adjusted for maternal age, parity, parental psychiatric history, gestational age, birth weight, congenital malformations, calendar period	No	Yes – dose–response by cigarette number (non-significant)	8

Kalkbrenner / 2012 [24]	No	No	No	DSM-IV-TR autism spectrum disorder diagnosis	ADDM surveillance with standardized abstraction of educational and clinical records	Mean ~8 years	Adjusted prevalence ratio (aPR)	0.90 (95% CI: 0.80–1.01)	Ever vs never	Maternal age, race/ethnicity, education, marital status, county population size, birth year, surveillance site	Yes – stratified by maternal race/ethnicity (e.g., PR 0.72 for non-Hispanic Black)	No	8
Lee / 2012 [25]	No	No	No	ASD (ICD-9 299; ICD-10 F84; DSM-IV 299), with and without intellectual disability	Multi-source case ascertainment from National Patient Register, VAL	4–17 years (diagnoses ascertained by 31 Dec 2007)	Adjusted odds ratio (conditional logistic regression)	Any smoking: aOR ≈ 1.03 (95% CI: 0.93–1.14); ≥10 cig/day: aOR ≈ 1.08 (95% CI: 0.93–1.26)	Ever vs never and dose–response categories	Maternal and paternal age, parity, parental education, occupational class, family income, maternal origin	Yes – ASD with and without intellectual disability	Yes – dose–response analysis	8

					database, psychiatric and habilitation services registers								
Maimburg / 2006 [26]	No	No	No	ICD-8 299.0 / ICD-10 F84.0 infantile autism	Danish Psychiatric Central Register diagnostic records	Diagnosis before age 10 (mean age ≈ 4.6 years)	Adjusted odds ratio (aOR)	0.9 (95% CI: 0.7–1.4)	Ever vs never	Adjusted for maternal and paternal age, maternal citizenship, birth weight, gestational age, Apgar score, congenital malformations, irregular fetal position	No	No	8
Nilsen / 2013 [27]	No	No	No	ICD-10 F84 autism spectrum disorder diagnosis	Norwegian Patient Registry	Median follow-up ≈ 7 years (median diagnosis	Adjusted odds ratio (aOR)	0.95 (95% CI: 0.78–1.15)	Ever vs never	Maternal age, parity, parental education, income, parental	Yes – stratified by timing (weeks 17 vs 30) and intensity	Yes – timing and intensity analyses (non-	8

					diagnostic data	age ≈ 6.7 years)				psychiatric history, child sex		significant)	
Mrozek-Budzyn / 2013 [28]	Yes – passive smoking assessed (aOR ≈ 2.57)	No	No	ICD-10 F84.0 / F84.1 autism spectrum disorder diagnosis	Clinical evaluation and confirmation by a child psychiatrist	Mean age at diagnosis ≈ 4.5 years	Adjusted odds ratio (aOR)	3.32 (95% CI: 1.12–9.82)	Ever vs never	Maternal age, parity, socioeconomic status, maternal education, birth complications, perinatal factors	Yes – stratified by sex (e.g., OR ≈ 3.17 for boys)	No	7
Tran / 2013 [29]	No	No	No	ICD-10 F84 autism spectrum disorder diagnosis	Finnish Hospital Discharge Register diagnostic records	Follow-up through 2007 (diagnosis typically age 8–14 years)	Adjusted odds ratio (aOR)	Overall ASD: aOR 1.0 (95% CI: 0.9–1.2); PDD: aOR 1.2 (95% CI: 1.04–1.5)	Three-category comparison (ref: none)	Maternal age, parity, socioeconomic status, gestational age, birth weight, parental psychiatric history	Yes – separate models for autism subtypes (childhood autism, Asperger's, PDD)	No	8
Berger/2021 [30]	Yes – ETS assessed	No	None	DSM (DDS validated	California DDS	Mean 5.24 years	aOR	0.73 (95% CI: 0.35–1.54) for cotinine-defined	Ever vs never (cotini	Maternal age, maternal education, maternal	Interaction by race: aOR 0.25 (95% CI:	No	8

	using continuous cotinine <3.08 ng/ml			diagnosis)	registry			smoking vs non-smoking	ne ≥3.08 vs <3.08)	race/ethnicity, insurance type, child sex, birth year/month	0.06–0.97) for Non-Hispanic Asian/Black/Other		
Caramaschi/ 2018 [11]	Yes – Partner smoking analysed as passive exposure	Yes – Partner smoking analysed as negative control	Yes – Partner smoking negative control; MR and methylation analyses	ICD-10 ASD diagnosis or reported diagnosis	Multiple sources: educational records, parental report, questionnaires, letters	Up to age 16 (ASD); traits up to 7.5–9.5 years	aOR / Beta	ASD diagnosis: aOR 0.84 (95% CI: 0.46–1.56); Social communication OR 1.23 (95% CI: 0.94–1.60)	Ever vs never	Sex, maternal age, parity, education, social class, financial difficulties, partner smoking, maternal depression	Yes – by trimester, by trait, by smoking heaviness	No	8
Costa / 2024 [31]	No	Yes – paternal smoking yes/no	None	Existing ASD diagnosis (clinic/ANDA)	Clinic/records confirmation per site	Mean ~6.5 years	OR / aOR	Crude OR 0.85 (95% CI: 0.34–2.09); no significant adjusted association	Ever vs never	Adjusted models for medicine use; smoking not significant	No	No	8

Grossi / 2018 [32]	No	No	Yes – sibling analysis (comparison with unaffected siblings)	DSM-5	Clinical diagnosis confirmed by child psychiatrist	Mean age 8.2 years	OR (unadjusted)	2.01 (95% CI: 0.54–7.37) for smoking during pregnancy vs none	Ever vs never	Not adjusted	Yes – by sibling vs control comparison	No	7
Cheslack-Postava/2021 [14]	No	No	No	Special education classification (ASD)	State special education registry linkage	Median age ~5.4 years	Prevalence ratio (adjusted)	0.96 (95% CI: 0.88–1.04)	Ever vs never	Maternal age, race, education, marital status, parity, birth weight, gestational age, infant sex	Stratified by maternal race/ethnicity and parity	No	8
Roigé-Castellví/2021 [33]	Yes – passive smoke at home and work assessed	Yes – paternal smoking during pregnancy reported	No	DSM-IV/DSM-5 equivalent criteria (clinical or registry-based)	Pediatric neurodevelopmental clinical assessments and	Median age 7.5 years	aRR (adjusted risk ratio)	1.09 (95% CI: 0.58–2.04)	Ever vs never	Maternal age, parity, education, BMI, alcohol use, socioeconomic status, maternal depression, paternal smoking	Yes – trimester-specific and by intensity	No	8

					medical records								
von Ehrenstein / 2020 [13]	No	No	Yes – sibling comparison performed	DSM-IV-TR	California DDS registry with validated ASD diagnosis	Mean ~7.2 years	Adjusted odds ratio (OR)	1.15 (95% CI: 1.04–1.26) (ever vs never); 1.55 (95% CI: 1.21–1.98) (≥20/day)	Ever vs never	Maternal age, race/ethnicity, education, parity, pregnancy complications, payer for delivery	Yes – stratified by presence/ absence of intellectual disability	Yes – significant dose–response with higher intensity	8
Yim/2022 [34]	No	Yes – paternal smoking reported separately	No	ICD-10 F84 (ASD)	NHIS claims data	Median follow-up 6.5 years	Hazard ratio (adjusted)	1.11 (95% CI: 1.06–1.17)	Ever vs never	Maternal age, parity, income, pre-pregnancy BMI, gestational age, birth weight, infant sex, parental psychiatric history	Yes – by infant sex, birth weight, gestational age	No	8
